# The difficulties of Adult ADHD management within a Community Mental Health Team

**DOI:** 10.1192/j.eurpsy.2024.999

**Published:** 2024-08-27

**Authors:** S. Haugh, H. Belay

**Affiliations:** Department of Psychiatry, Connolly hospital, Dublin, Ireland

## Abstract

**Introduction:**

ADHD is a neurodevelopmental disorder characterised by: inattention, hyperactivity and impulsivity.Diagnosis of ADHD in adults is complex, owing to the need for retrospective evidence that symptoms began in childhood as well as the high rates of comorbid mental health conditions. There are no public specialized clinics for adults with ADHD in Ireland. In their absence, referrals are sent to general adult psychiatry.

**Objectives:**

An audit of standards of care received by patients with ADHD against those set by the NICE guidelines.

**Methods:**

Care received pre and 8 weeks post MDT (multi-disciplinary team) educational session. Inclusion criteria: existing adult community mental health team (CMHT) patients with a diagnosis of ADHD. Recommendations as per NICE guideline used for assessment: Specialist MDT team input, OT/ Psychology input, MDT review of reports, Specialist consultant with training in diagnosis and treatment, Diagnosis based on structured assessment e.g. DIVA, Detailed psychiatric assessment, Physical health monitoring before commencing treatment (e.g. ECG), Ongoing physical health monitoring (BP, HR, weight), Patient regularly attending follow up

**Results:**

There were 7 patients with diagnosed ADHD attending the CMHT, 4 male, 3 female aged 19-42yo. 4 patients were diagnosed privately (average age at diagnosis 31yrs). 2 were diagnosed by CAMHS. And 1 was diagnosed by primary care psychology (age 27). 8 weeks following MDT meeting; 2 patients had been commenced on ADHD medication. Those on the wait list for OT/psychology remained on the wait list.

**Conclusions:**

ADHD is a specialised area which requires a specialist MDT led by a consultant with expertise in diagnosis and treatment. As evidenced by this audit, despite the best efforts of adult psychiatric services, teams are not sufficiently resourced to meet the needs of adults with ADHD and fall short of the expected standards of care.

**Disclosure of Interest:**

None Declared

